# Supplementary material for: RNA structure prediction using positive and negative evolutionary information
Source: PLoS Comput Biol. 2020 Oct 30;16(10):e1008387. doi: 10.1371/journal.pcbi.1008387 (PMC7657543; doi:10.1371/journal.pcbi.1008387)
Supplement: S1 File — Includes alignments, and source code. (GZ) [file pcbi.1008387.s008.tar.gz › supplemental_material/src/rscape_v1.5.4.tar.gz!/rscape_v1.5.4/lib/infernal/infernal-1.1.2/documentation/userguide/Figures/cm_graph.pdf]

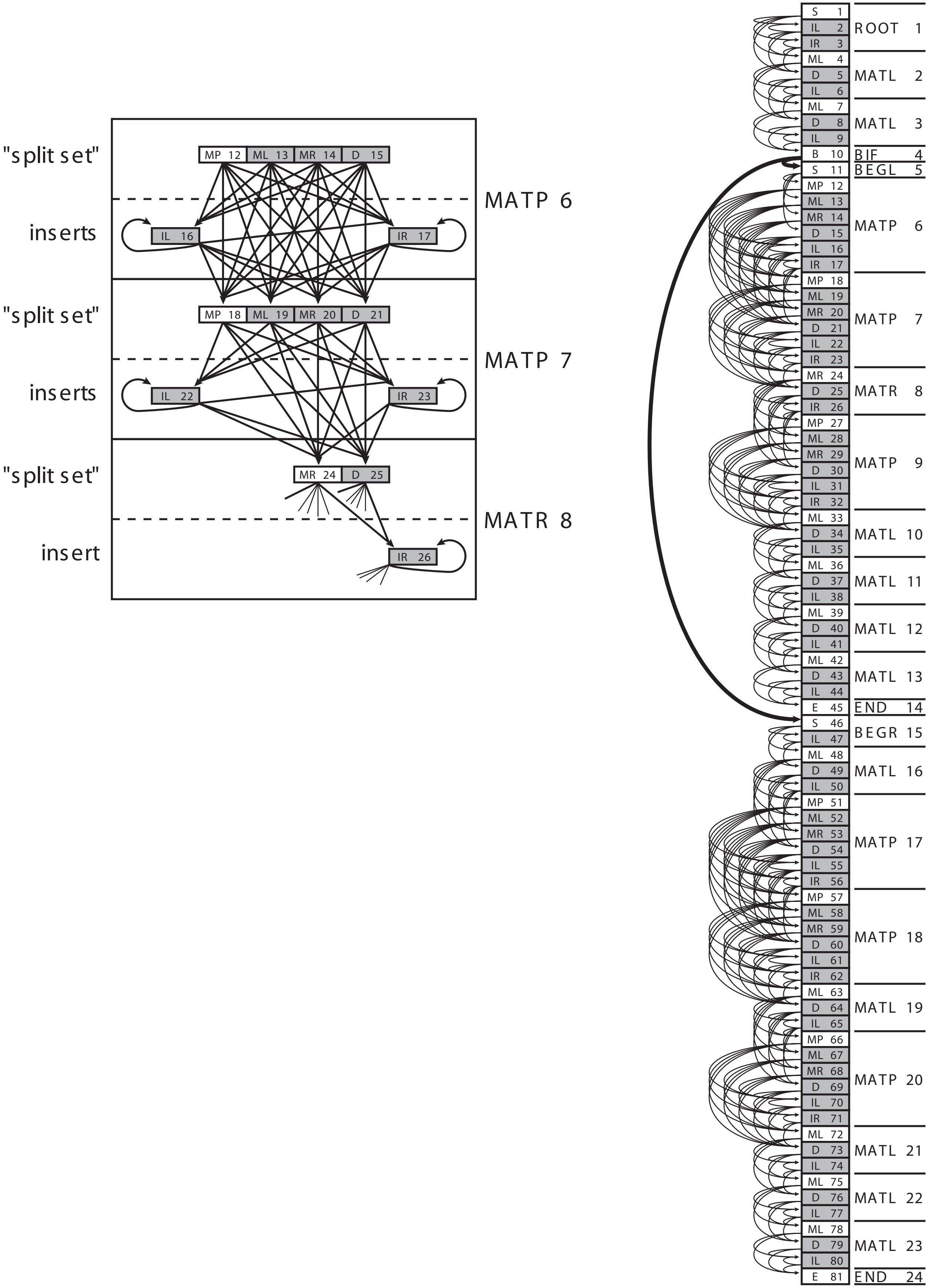
"split set"
MP 12
ML 13
MR 14
D 15
MATP 6
inserts
IL 16
IR 17
"split set"
MP 18
ML 19
MR 20
D 21
MATP 7
inserts
IL 22
IR 23
"split set"
MR 24
D 25
MATR 8
insert
IR 26
S 1
IL 2
IR 3
ROOT 1
ML 4
D 5
IL 6
MATL 2
ML 7
D 8
IL 9
MATL 3
B 10
BIF 4
S 11
BEGL 5
MP 12
ML 13
MR 14
D 15
IL 16
IR 17
MATP 6
MP 18
ML 19
MR 20
D 21
IL 22
IR 23
MATP 7
MR 24
D 25
IR 26
MATR 8
MP 27
ML 28
MR 29
D 30
IL 31
IR 32
MATP 9
ML 33
D 34
IL 35
MATL 10
ML 36
D 37
IL 38
MATL 11
ML 39
D 40
IL 41
MATL 12
ML 42
D 43
IL 44
MATL 13
E 45
END 14
S 46
IL 47
BEGR 15
ML 48
D 49
IL 50
MATL 16
MP 51
ML 52
MR 53
D 54
IL 55
IR 56
MATP 17
MP 57
ML 58
MR 59
D 60
IL 61
IR 62
MATP 18
ML 63
D 64
IL 65
MATL 19
MP 66
ML 67
MR 68
D 69
IL 70
IR 71
MATP 20
ML 72
D 73
IL 74
MATL 21
ML 75
D 76
IL 77
MATL 22
ML 78
D 79
IL 80
MATL 23
E 81
END 24
